# Paraovarian cyst with associated ovarian torsion: The importance of early diagnosis

**DOI:** 10.31053/1853.0605.v80.n4.40830

**Published:** 2023-12-26

**Authors:** Deborah Desiree Coelho Marra, Bruna Suda Rodrigues, Gabriel Miura, Élcio Roberto Duarte, Márcio Luís Duarte

**Affiliations:** 1 Faculdade de Ciências Médicas de Santos Santos SP Brasil; 2 Irmandade da Santa Casa de Misericórdia de Santos Santos SP Brasil; 3 Universidade de Ribeirão Preto Campus Guarujá Guarujá SP Brasil

**Keywords:** ultrasonography, tomography, x-ray computed, ovarian torsion, ultrasonografía, tomografía computarizada por rayos x, torsión ovárica, ultrassonografia, tomografia computadorizada por raios x, torção ovariana

## Abstract

The paraovarian or paratubarian cysts are both situated in the broad ligament between the ovary and fallopian tube. The diagnosis of adnexal torsion is challenging since both symptoms and physical examination are nonspecific. In most cases, the patient presents abdominal pain, followed by nausea and vomiting. Imaging tests, such as ultrasound, are very useful to elucidate the cause of the symptoms in those patients.

## Resumen

Los quistes paraováricos o paratubáricos están situados en el ligamento ancho entre el ovario y la trompa de Falopio. El diagnóstico de torsión anexial es un desafío ya que tanto los síntomas como el examen físico son inespecíficos. En la mayoría de los casos, el paciente presenta dolor abdominal, seguido de náuseas y vómitos. Las pruebas de imagen, como la ecografía, son de gran utilidad para dilucidar la causa de los síntomas en estos pacientes.

## Resumo

Os cistos paraovarianos ou paratubarianos estão situados no ligamento largo entre o ovário e a tuba uterina. O diagnóstico de torção anexial é desafiador, pois os sintomas e o exame físico são inespecíficos. Na maioria dos casos, o paciente apresenta dor abdominal, seguida de náuseas e vômitos. Os exames de imagem, como a ultrassonografia, são muito úteis para elucidar a causa dos sintomas nestes pacientes.

**KEY CONCEPTS**

**What is known on the subject?**

The paraovarian or paratubarian cysts are both situated in the broad ligament between the ovary and fallopian tube. The torsion mechanism occurs when adnexal structures such as uterine tubes, ovaries, and paraovarian cysts rotate around the pelvic infundibulum, compromising local vascularization and potentially progressing to dysfunction, ovarian ischemia, and infertility. The delay in diagnosis reflects in the failure of early surgical approach, which may trigger to ovarian or tubal necrosis, major dysfunction and infertility.

**What does this work contribute?**

This article could contribute to the discussion of the early treatment of ovarian torsion caused by paraovarian cyst. In our case, the 26-year-old female patient was treated with laparoscopic approach with right oophorectomy and salpingectomy was immediately performed after the ultrasound. On the second postoperative day she was discharged.

**Divulgación**

Un quiste paraovárico se encuentra en el ligamento ancho entre el ovario y la trompa de Falopio. A veces, este quiste hace que los vasos ováricos se tuerzan, lo que provoca un dolor abdominal intenso. Los factores de riesgo comunes incluyen cirugía pélvica previa, quistes ováricos, inducción de la ovulación y embarazo (especialmente en el primer trimestre). En caso de retraso diagnóstico, la falta de vascularización provocará isquemia ovárica, seguida de necrosis e infertilidad. La ultrasonografía es un examen costo-efectivo que permite el diagnóstico precoz, direccionando el tratamiento adecuado, evitando las complicaciones de la enfermedad.

The paraovarian or paratubarian cysts are both situated in the broad ligament between the ovary and fallopian tube.1 They may show up as an acute abdomen condition^1^. Despite their low incidence, these cysts are present in three out the 20% of all adnexal masses reported in different age groups^2^. Premenopausal women constitute a predominantly affected group^3^.

The diagnosis of adnexal torsion is challenging since both symptoms and physical examination are nonspecific. In most cases, the patient presents abdominal pain, followed by nausea and vomiting. These symptoms with leukocytosis and fever are frequently found on this condition, but they are also present in a variety of differential pathologies, such as acute appendicitis, severe ovarian cyst, ectopic pregnancy or pelvic inflammatory disease.1 Those symptoms may also be related to possible complications of the cyst, such as torsion in its own axis, rupture, and hemorrhage^3^.

The torsion mechanism occurs when adnexal structures such as uterine tubes, ovaries, and paraovarian cysts rotate around the pelvic infundibulum, compromising local vascularization^3^. The common risk factors include previous pelvic surgery, ovarian cysts, induction of ovulation and pregnancy (especially in the first trimester). Nonetheless, no risk factors are observed in up to 69% of patients with surgically confirmed torsion^3^.

The presence of ovarian edema is the main marker to aid the diagnosis of adnexal torsion. In the absence of this finding, when both ovaries maintain their usual size and locations on ultrasound, CT or MRI, the diagnostic hypothesis of torsion is promptly ruled out^3^. Stromal edema associated with venous congestion caused by torsion may displace fluid to the peripheral follicles, resulting in the "pearl necklace" sign, observed in 71.4% of the cases submitted to Doppler ultrasonography and in 85.7% of the cases submitted to MRI^4^.

A laparoscopic approach is preferred and in complicated situations, such as the identification of deep adhesions^2^. The delay in diagnosis reflects in the failure of early surgical approach, which may trigger to ovarian or tubal necrosis, major dysfunction and infertility^3^.

A 26-year-old woman, in remote puerperium – cesarean delivery 60 days ago – reports pain of high intensity for 12 hours in the right iliac fossa, associated with inappetence, nausea and four episodes of vomiting. The patient denies improvement of pain using tramadol.

On physical examination, she presented a painful abdomen on deep palpation of the right iliac fossa, presence of bowel sounds and negative Blumberg sign. Leukocytosis was present in the hemogram test (12260 /mm³).

Abdominal CT shows a retro-uterine cyst (Fig 1; white arrow). The transvaginal ultrasonography diagnosed a right paraovarian cyst near to the right ovary which shows reduced vascularization in comparison to the left ovary, inferring ovarian torsion (Fig 2; white arrow).

**Figure N° 1.**
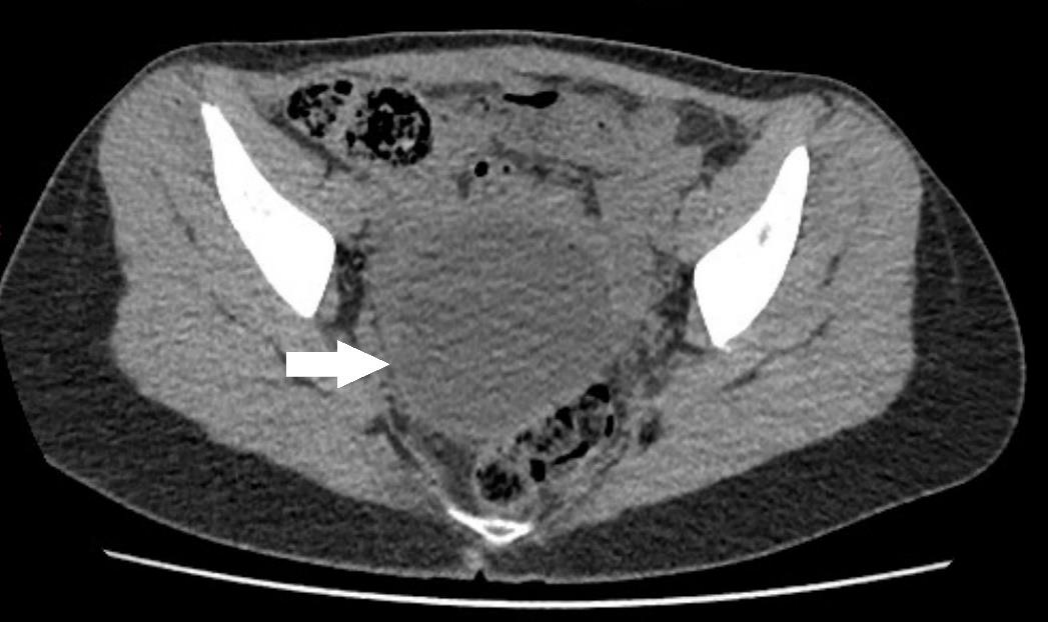
Non-contrast CT scan of pelvis showing cystic image in the retro-uterine region on the right (white arrow).

**Figure N°2.**
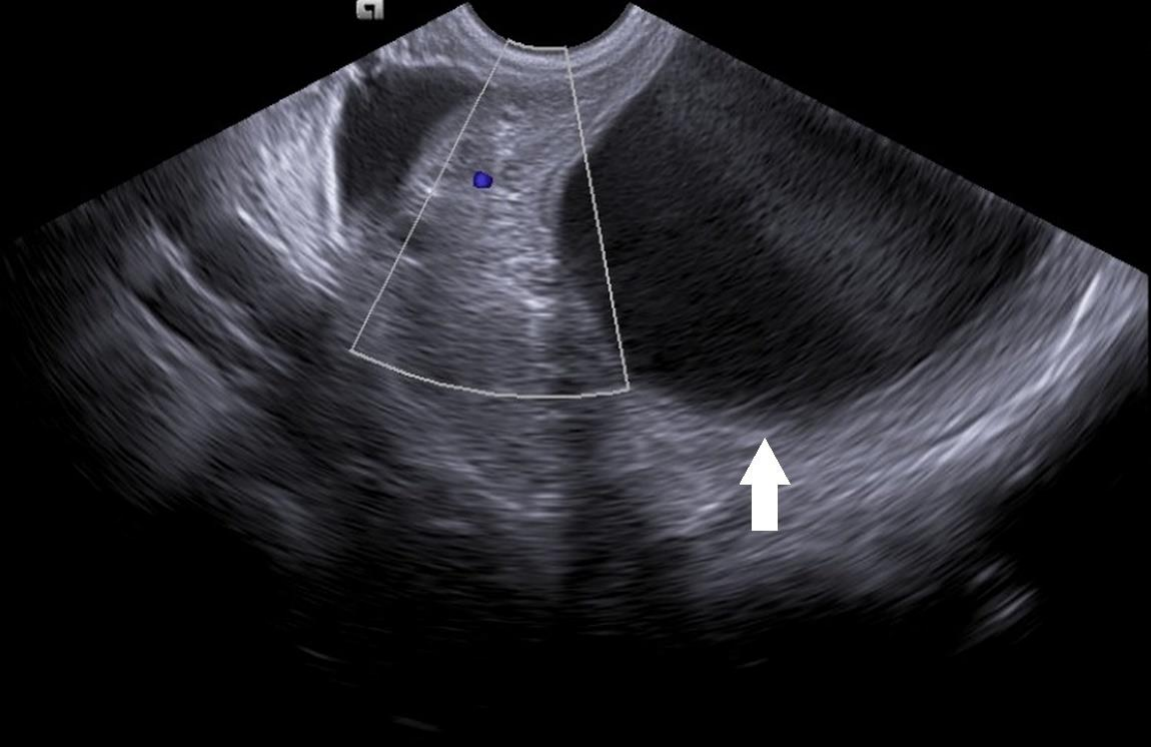
Transvaginal pelvic ultrasound showing right ovary with reduced vascularization on a Doppler study and the paraovarian cyst (white arrow).

A laparoscopic approach with right oophorectomy and salpingectomy was immediately performed after the ultrasound. In one day of evolution, the patient is painless, walking, with elimination of flatus and good nutrition, without pain on abdominal palpation, being discharged from hospital on the second postoperative day with a follow-up orientation to return to gynecology department.
